#  Family Analysis of Linkage and Association of HLA-DRB1, CTLA4, TGFB1, IL4, CCR5, RANTES, MMP9 and TIMP1 Gene Polymorphisms with Multiple Sclerosis 

**Published:** 2011

**Authors:** O.Yu. Makarycheva, E.Yu. Tsareva, M.A. Sudomoina, O.G. Kulakova, B.V. Titov, O.V. Bykova, N.V. Gol’tsova, L.M. Kuzenkova, A.N. Boiko, O.O. Favorova

**Affiliations:** Russian Cardiology Scientific and Production Center; Russian State Medical University; Scientific Center of Children Health, Russian Academy of Medical Sciences

**Keywords:** functional genomics, human, multiple sclerosis, genotyping, *CCR5 * gene, *CTLA4 * gene, HLA*-DRB1* gene, *IL4* gene, *MMP9* gene, *RANTES* gene, *TGFB1* gene, *TIMP1* gene, allelic polymorphism, TDT, AFBAC

## Abstract

Multiple sclerosis (MS) is a chronic inflammatory autoimmune disease of the central nervous system (CNS). Proteins of the immune system, as well as proteins that are involved in the infiltration of activated immune cells in the CNS, play an important role in the pathogenesis of MS. We investigated the association and linkage with MS of the following immune-system genes polymorphisms: HLA-*DRB1*,*CTLA4*,*TGFB1*,*IL4*,*CCR5 *and*RANTES*, as well as of the matrix metalloproteinase 9 (*MMP9*) and tissue inhibitor of metalloproteinase  1 (*TIMP1*) genes polymorphisms. For this purpose we used the transmission disequilibrium test (TDT). The group investigated was comprised of 100 nuclear families of Russian ethnicity, each consisting of an affected offspring and his nonaffected parents. It was found that HLA-DRB1*15**allele**andMMP9*-1562C allele were transmitted from healthy heterozygous parents to affected children more frequently than alternative alleles (*p  *=  0.02 and*p  *=  0.04, respectively). Another family-based method, AFBAC (affected family-based control), showed MS association with HLA-DRB1*15, but not with theMMP9*-1562C allele.

##  INTRODUCTION 

Multiple sclerosis (MS) is a severe inflammatory disease of the central nervous system (CNS) which typically develops in the young adults and subsequently leads to disability. This disease has a complex etiology with both genetic and environmental factors [1]. The ways MS is inherited are typical for polygenic diseases; their development is conditioned by the joint contribution of a number of polymorphic genes [2]. The elicitation of the genetic risk factors for MS may help shed light on the mechanisms underlying the pathogenesis of this disease and open new possibilities for its prevention and treatment. In spite of the large number of studies that have investigated the genetics of MS, the search for MS-associated genes remains a challenge. This is due to the nature of the disease, for which genetic heterogeneity is typical, particularly in different ethnic groups, as well as the absence of a main gene. On the other hand, the search for the risk factors for MS is made more difficult by the limitations of the major analysis approaches. The MS genome linkage analysis has yielded little information because of its low sensitivity [3]. When analyzing genetic associations with MS using the case–control method, there is a low reproducibility of the results: a factor related to the ethnic heterogeneity of the healthy and affected groups under consideration and the influence of environmental factors [4].

The methods that use a family analysis of associations allow to eliminate or to minimize the influence of the ethnic heterogeneity of groups of patients and unrelated healthy controls, as well as the effect of environmental factors [5]. One such method is the transmission disequilibrium test, TDT [6], which is based on the analysis of marker allele or haplotype transmission from heterozygous parents to affected children. The TDT method has already been used to analyze the linkage and association of the alleles of a number of candidate genes with MS among various ethnicities [7–10], including Russians (our studies [11, 12]). This method is now being used not only to analyze the contribution of individual genes to the MS development, but also as a tool in a full genome search [13–15]. Family data have also been used to carry out the association analysis using the affected family-based control (AFBAC) method. According to this data, the control group is composed of a set of alleles from healthy parents which were not transmitted to affected children (one allele from each parent) [16]. This method was used to analyze MS susceptibility in Italy [17, 18], Great Britain [19], Belgium [20], and France [21]. Each of these methods of family analysis has advantages and drawbacks. Thus, AFBAC is a more powerful method as compared with TDT, while TDT makes it possible to completely eliminate the population stratification effects [22].

In this study, we analyzed MS linkage and association of HLA *-DRB1, CTLA4, TGFB1, IL4, CCR5, RANTES, MMP9, * and * TIMP1 * genes in ethnic Russians on the basis of family data using the TDT and AFBAC methods.

Numerous data suggest the involvment of these genes in the immunopathogenesis of MS as an autoimmune disease [2]. A repeatedly confirmed fact is that particular alleles (depending on the population ethnicity) of the HLA *-DRB1 * gene class II are involved in the development of MS. This gene encodes the β-chain of the heterodimer, which presents the antigen to CD4+ T-lymphocytes. Our study also includes the *CTLA4* gene encoding the cytotoxic T-lymphocyte antigen  4 (CTLA4 or CD152) – the T-lymphocytes costimulation receptor, which is an important negative regulator of the T cells activity and participates in the maintenance of the peripheral T  cell tolerance [23].

Cytokines are believed to play the key role in the development and regulation of the autoimmune inflammatory process that is typical of MS. In addition to the data obtained by the analysis of MS linkage and association with alleles of proinflammatory cytokine genes [12], the genes of anti-inflammatory cytokines TGFβ1 and IL-4 – were also considered in this study. Cytokine TGFβ1 is secreted by numerous cell types, including regulatory T- lymphocytes, astrocytes, and endothelial cells; while cytokine IL-4 is secreted mainly by activated Th2 -cells. These cytokines can be detected in the brain tissues at the remission stage; their level being reduced upon active progressive multiple sclerosis [24].

The key stage in the development of the immunopathological process upon MS is the disturbance of the hematoencephalic barrier and T and B cells penetration into the CNS. The stimulation and direction of migration of different cell classes is to a large extent determined by chemokines. Our study includes genes of RANTES (Regulated on Activation Normal T cells Expressed and Secreted) chemokine, which is an attractant for lymphocytes and monocytes, and of its receptor CCR5. The levels of RANTES and  CCR5 sharply increase on lymphocytes, macrophages, and microglias in the demyelination lesions upon MS acute attack [25].

The penetration of immune cells into the CNS is accompanied by the type  IV collagen degradation, which is the extracellular matrix major constituent. The matrix metalloproteinases (MMPs) play the key role in this barrier penetration. The MMPs are involved in various stages of MS pathogenesis: they participate in the local damaging of the hematoencephalic barrier and perivascular lymphocytes infiltration, in the damaging of myelin sheath and in the formation of the demyelination lesions and axonal death [26]. One of the major metalloproteinases, MMP9, is expressed by perivascular mononuclear cells of white matter and, together with other MMPs, is associated with monocytes and astrocytes in the demyelination lesions.

MMP activity is controlled by tissue inhibitors of matrix metalloproteinases (TIMPs), while the TIMP1 level in the cerebrospinal fluid of MS patients was found to be decreased [26]. Considering these data, the *MMP9* and   *TIMP1 * genes were included in our study.

The genomic typing of 18  allelic groups of the HLA- *DRB1 * gene and the following single-nucleotide polymorphisms (SNPs) 49A>G of the *CTLA4* gene; –509C>T ofthe *TGFB1 * gene; –590C>T of *IL4* gene; –403G>A of the *RANTES * gene; –1562C>T of the *MMP9 * gene; 372C>T of the *TIMP1 * gene; as well as of the deletion-insertion polymorphism *CCR5 * (w→d) (“wild type” → deletion of 32  bp), was carried out for Russian MS patients and their healthy parents, followed by the analysis of genetic predisposition to MS using the TDT and AFBAC methods. Selection of the polymorphic regions for the analysis proceeded from the data on their influence on the level and/or activity of the encoded proteins Thus, the functional roles of the *DRB1 * gene alleles in antigen presentation and the consequences of deletion in the *CCR5 * gene resulting in the production of the CCR5 inactive protein are well known. With regard to the SNPs analysis, rare alleles of the *TGFB1* , *IL4* , * RANTES, * and * MMP9 * genes are associated with the enhanced production of the protein [27–30], whereas the expression of the encoded protein on the cell surface is increased in the carriers of the allele A of the *CTLA4 * gene [31]. SNP 372C>T of the *TIMP1 * gene is the only exception with no data available.

##  EXPERIMENTAL 

**The object of investigation**

The study was performed using peripheral blood samples obtained from members of 104 nuclear families, each of them comprising MS patients and their healthy parents. The blood samples were collected at the Research Center of Children Health *, * Russian Academy of Medical Sciences, and at the Multiple Sclerosis Moscow City Center . The MS diagnosis was done according to McDonald’s criteria [32]. There were 46 male patients and 58 female ones; all patients experienced the onset of MS before the age of 35. The mean MS onset age was 18  ±  8 years. The disease had a relapsing–remitting course in 102 patients and a primary progressive course in two patients. All patients and their healthy relatives were residents of the Moscow region and belonged to Russian ethnicity. All families provided their informed consent for participation in the study.

**DNA extraction and genotyping**

Genomic DNA was extracted from mononuclear blood cells using a phenol–chloroform mixture according to the standard procedure [33].

*Table 1* lists the polymorphic regions of the analyzed genes, PCR-based genotyping methods, and the primers used. Typing of the HLA *-DRB1 * gene was carried out by allele-specific PCR, in accordance with the recommendations of the manufacturer of the kit (AO DNA Technology, Russia) used to identify the allele groups corresponding to serological specificities from DR1 to DR18.

**Statistical analysis**

Linkage and association of the genes with MS was studied using TDT [6] with χ ^2 ^ criterion. Haploview  3.32 free software was used for analysis of bi-allelic polymorphism, and FBAT [35] was used for analysis of multi-allelic polymorphism of the HLA- *DRB1 * gene and polymorphism of the *TIMP1 * gene located on the *X* -chromosome. We analyzed the transmission of gene alleles from parents to affected children in families with at least one heterozygous parent. The difference between the frequencies of transmitted and non-transmitted alleles was considered significant at χ ^2^  >  3.8 *(p *  < 0.05). In order to analyze MS association with alleles of the studied genes using the AFBAC method, the control group was made up of the alleles of both parents which were not transmitted to the affected children. The probability value ( *p* ) was assessed with the two-sided Fischer’s exact test using GraphPAD InStat 1.12a software.

 The discrepancy in the observed distribution of genotype frequencies in the groups of patients and their healthy parents from the Hardy–Weinberg equilibrium was analyzed by the expectation maximization algorithm, using Haploview  3.32. 

##  RESULTS AND DISCUSSION 

All the members of the 104  nuclear families were genotyped for the polymorphisms shown in *Table 1.* Four families, for which the paternity was not confirmed by the results of genotyping, were excluded from further analysis. Using Haploview  3.32, we showed that the genotype frequency distributions in MS patients and their parents were in Hardy–Weinberg equilibrium ( *р * < 0.05).

TDT was used to obtain the χ ^2^ values characterizing the difference between the observed frequencies of inheritance of alleles of the HLA *-DRB1, CTLA4, TGFB1, IL4, CCR5, RANTES, MMP9, * and * TIMP1* genes by affected children from 100  nuclear families from the frequencies expected in the absence of association between the allele and the disease. The χ ^2 ^ value was used to calculate the *p * value ( *Table 2* ). We detected a significant linkage/association between MS and the HLA *-DRB1* *15 (χ ^2^   =  5.7, * p  * =  0.02) and *MMP9* *(–1562)С (χ ^2^   =  4.1, *p  * =  0.04) alleles. For the remaining polymorphic regions, no significant results were obtained (χ ^2^  <  3.8, *p *  >  0.05). TDT did not detect the linkage/association of any studied polymorphism with MS in male and female groups separately.

*Table 3* lists the results of the AFBAC analysis of MS association with the examined polymofphic alleles. By comparing the allele frequency in affected children with that in the control group consisting of the alleles belonging to their mothers and fathers and not transmitted to their children, we detected a significant MS association only with the HLA *-DRB1* *15 allele ( *p * = 0.02), but not with the alleles of other genes.

 Despite the fact that the family-based association analysis has a number of advantages over the population-based analysis, such investigations remain relatively rare around the world. This is particularly true for Russia due to the complexity of collecting nuclear family data. Along with the issue of incomplete families, cases of false paternity are of considerable quantity. Thus, we had to exclude four families out of the initial 104  families in the study. 

**Table 1:** Polymorphisms of the analyzed genes, genotyping methods, and primers used

Gene	Polymorphism	SNP ID	Analysis method (reference)	PCR primers (restrictase used)
HLA*-DRB1*	Allele 01–18 groups, corresponding to DR1–DR18 specificities	–	SSP-PCR	From the kit for amplification of HLA-* DRB1 *(AO DNA Technology, Russia)
*CTLA4*	SNP 49A>G (17Thr → Ala)	rs231775	PCR RFLP [34]**	5’-AAGGCTCAGCTGAACCTGGT and 5’-CTGCTGAAACAAATGAAACCC[BstEII]
*TGFB1*	SNP -509C>T	rs1800469	SSP-PCR	5’-GGGCAACAGGACACCTGAA-3’(SSP T),5’-GGGCAACAGGACACCTGAG-3’ (SSP C), and 5’-AAGGCATGGCACCGCTTCTG-3’ (common forward primer)
*IL4*	SNP -590C>T	rs2243250	SSP-PCR	5’-CTAAACTTGGGAGAACATTGTC-3’ (SSP C), 5’-CTAAACTTGGGAGAACATTGTT 3’ (SSP T), and 5’-AGTACAGGTGGCATCTTGGAAA-3’ (common reverse primer)
*CCR5*	(w → d) (”wild type” → deletion of 32 bp)	–	PCR	5’-AGGTCTTCATTACACCTGCAGC-3’ and 5’-CTTCTCATTTCGACACCGAAGC-3’
*RANTES*	SNP -403G>A	rs2107538	SSP-PCR	5’-CCATGGATGAGGGAAAGGAGG-3’ (SSP G), 5’-CCATGGATGAGGGAAAGGAGA-3’ (SSP A), and 5’-AGGGAAGGGGTCCTCCTCAG-3’ (common reverse primer)
*MMP9*	SNP -1562C>T C	rs3918242	PCR RFLP	5’-GCCTGGCACATAGTAGGCCC-3’ and 5’-CTTCCTAGCCAGCCGGCATC-3’ [SphI]
*TIMP1*	SNP 372C>T	rs4898	SSP-PCR	5’-CTGTTCAGGGAGCCACG-3’ (ASP SSP C), 5’-CTGTTCAGGGAGCCACA-3’ (SSP T), and 5’-AGCGAGGAGTTCTCATTGCT-3’ (common forward primer)

* All SNP positions are presented relative to transcription start sites, with the exception of CTLA4 49A>G, where 49 is the position relative to the translation start site.

** The reference is given for the case when the technique described was used.

. SSP–site-specific primer; RFLP–restriction fragment length polymorphism.

The genes whose participation in the development of MS was studied in this work can be divided into two groups, with respect to the extent they were previously investigated in Russians. The HLA *-DRB1, CTLA4, TGFB1, * and  *CCR5 * genes can be referred to the first group; we had previously analyzed MS association with the polymorphism of the above genes using the case–control method with unrelated individuals as controls. The second group included the *IL4, RANTES, MMP9* , and *  TIMP1* genes, which had not previously been studied in details.

The replication (validation) of the data on the association of a particular gene with the disease on independent samples is now considered as a necessary condition for the results to be accepted by the scientific community. This requirement was thoroughly satisfied in our study for the genes from the first group. The MS association with *DRB1* *15 HLA class  II allele in Russians, which was revealed by TDT and  AFBAC, had been shown earlier in population-based studies [36, 37]. On the other hand, we observed no MS association with alleles of the *CTLA4, TGFB1,* and * CCR5 * genes neither while carrying out the family-based analysis in this study nor while carrying out the case-control analysis on independent groups of unrelated individuals [37–39].

We had previously used the TDT method to show the MS linkage/association of *DRB1* *15 HLA class II allele in children and adolescents (so-called juvenile MS, with onset below the age of 15) [11]. Since only DNA samples obtained from 39  nuclear families were available, we simulated the analysis of the bi-allelic locus and compared the carriers of the *DRB1* *15 allele with allele non-carriers: i.e., the carriers of all other alleles of the *DRB1 * gene. In the present study, we first analyzed the MS linkage and association with all the alleles of multi-allele polymorphism of the HLA- *DRB1 * gene in Russians and confirmed MS association and linkage with the *DRB1* *15 allele, regardless of the age of onset.

The data on MS association with polymorphism of *CCR5* (w→d) [40–42] and  SNP 49A>G of *CTLA4 * in other Caucasians are controversial [43–45], whereas no MS association with SNP –509C>T of *TGFB1 * has been observed [46–48]. As for the *DRB1 * gene, it appears to be the major genetic risk factor for MS in all Caucasians, although MS associations with other allelotypes, rather than *DRB1* *15, have been detected in some populations in the Mediterranean [2].

**Table 2:** Transmission of polymorphous alleles of HLA *-DRB1,CTLA4, TGFB1, IL4, CCR5, RANTES, MMP9* , and *TIMP1 * genes from healthy heterozygous parents to children with multiple sclerosis in100 nuclear families (analysis by TDT)*

Gene	Allele	Number of heterozygous parents	Transmitted, cases	Non-transmitted, cases	χ^2^	p
*DRB1*	01	35	22	13	2.3	> 0.05
	04	34	16	18	0.2	> 0.05
	07	36	18	18	0.0	> 0.05
	08	12	4	8	1.3	> 0.05
	11	53	24	29	0.5	> 0.05
	13	37	18	19	0.1	> 0.05
	15	70	45	25	5.7	0.02
	16	11	3	8	2.3	> 0.05
	17	46	20	26	0.8	> 0.05
*CTLA4*	A	104	51	53	0.04	> 0.05
	G		53	51		
*TGFB1*	C	101	48	53	0.3	> 0.05
	T		53	48		
*IL4*	C	69	32	37	0.4	> 0.05
	T		37	32		
*CCR5*	w	39	20	19	0.03	> 0.05
	d		19	20		
*RANTES*	G	63	31	32	0.1	> 0.05
	A		32	31		
*MMP9*	C	48	31	17	4.1	0.04
	T		17	31		
TIMP1**	C	49	28	21	0.5	> 0.05
	T		21	28		

* Data for the *DRB1* *09, *10, *12, and *14  alleles, the number of carriers of which among healthy parents is ≤  5 (2.5%), are insignificant and are not listed in *Tables  2 *  and   *3* .

** Since the TIMP1 gene is located on the *X* -chromosome, we considered the transmission of alleles to affected children only from heterozygous mothers.

The absence of individual MS association with polymorphisms in the *CTLA4* , *TGFB1* , and   *CCR5 * genes does not exclude their possible role as genetic risk factors for the disease, in combination with several other alleles/genotypes. Indeed, the alleles CTLA4* 49G, TGFB1* (–509)С, and   *CCR5* *d are the parts of MS-predisposing bi- and tri-allelic combinations with alleles of other genes found using the APSampler algorithm [37]. Combinations of alleles including the *CTLA4* and   *TGFB1 * genes that are associated with MS were detected in other studies [2]. The question of whether the association of allelic combinations with polygenic diseases can be determined either by the additive contribution of separate genes or by gene-gene interactions remains open.

Among the genes first time investigated using the TDT method in Russian MS patients, nonrandom transmission of the MMP9*(–1562) C allele from healthy parents to the affected child was shown. The results obtained for other Slavic populations (in Serbia [49] and the Czech Republic [50]) showed a significant decrease in T-allele frequency in MS patients as compared with healthy individuals and are in agreement with our data that allele C participates in the development of MS. However, these results were not confirmed with the AFBAC method in our study, whereas T  allele in Poles [51] acts as a MS predisposing allele. Thus, the issue of the participation of the *MMP9 * gene in the appearance of MS predisposition requires additional studies.

**Table 3:** Family-based analysis of MS association with alleles of HLA *-DRB1, CTLA4, TGFB1, IL4, CCR5, RANTES, MMP9* , and *TIMP1 * genes in  100 nuclear families carried out using the AFBAC method

Gene	Allele	Number (%) of alleles in MS patients (*n* = 200)	Number (%) of alleles that were not transmitted to affected children by parents (*n* = 200)	p
*DRB1*	01	25 (12.5)	6 (8)	> 0.05
	04	22 (11.0)	25 (12.5)	> 0.05
	07	22 (11.0)	22 (11.0)	> 0.05
	08	5 (2.5)	9 (4.5)	> 0.05
	11	25 (12.5)	30 (15)	> 0.05
	13	21 (10.5)	22 (11)	> 0.05
	15	50 (25)	30 (15)	0.02
	16	3 (1.5)	8 (4)	> 0.05
	17	22 (11)	28 (14)	> 0.05
*CTLA4*	A	113 (57)	103 (52)	> 0.05
	G	87 (43)	97 (48)	
*TGFB1*	C	132 (66)	137 (69)	> 0.05
	T	68 (34)	63 (31)	
*IL4*	C	158 (79)	165 (83)	> 0.05
	T	42 (21)	35 (17)	
*CCR5*	w	179 (90)	178 (89)	> 0.05
	d	21 (10)	22 (11)	
*RANTES*	G	163 (82)	164 (82)	> 0.05
	A	37 (18)	36 (18)	
*MMP9*	C	120 (60)	105 (53)	> 0.05
	T	80 (40)	95 (47)	
*TIMP1*	C	88 (56)	80 (55)	> 0.05
	T	68 (44)	65 (45)	

We did not reveal MS association with polymorphisms of the *IL4, RANTES* , and * TIMP1 * genes with the two methods of family-based analysis. According to published data SNP –590C>T of the *IL4* gene is associated with MS in Germans, particularly in females [52]. In Spaniards, SNP 33C>T of the *IL4 * gene is associated with MS, demonstrating a total linkage disequilibrium with SNP -590C>T of this gene [53]. The results of a study carried out in Iranians are in agreement with our data on the absence of MS linkage/association with the 33C>T region of the *IL4 * gene [54]. Association of *RANTES* SNP –403G>A with MS remains insufficiently studied; however, association of this polymorphism with MS was observed in Caucasians in the single study found, [55]. Data concerning the analysis of the 372C>T region of the *TIMP1 * gene has not been published.

##  CONCLUSIONS 

In this study, we used the methods of family-based analysis of linkage and/or association, which allow to eliminate or minimize the effects of a possible ethnic heterogeneity of the studied sample in the obtained results. The role of the HLA *-DRB1, CTLA4, TGFB1, IL4, CCR5, RANTES, MMP9* , and *TIMP1 * genes polymorphisms in MS development in ethnic Russians was analyzed. The data obtained by TDT are evidence of MS linkage/association with the *DRB1* *15 and   *MMP9* *(–1562)С alleles; association of *DRB1* *15 with the disease was confirmed by the AFBAC method. The present study shows the efficiency of the family-based approach in the study of polygenic diseases.
